# LAMTOR2-Mediated Modulation of NGF/MAPK Activation Kinetics during Differentiation of PC12 Cells

**DOI:** 10.1371/journal.pone.0095863

**Published:** 2014-04-21

**Authors:** Bettina Thauerer, Paul Voegele, Natascha Hermann-Kleiter, Nikolaus Thuille, Mariana E. G. de Araujo, Martin Offterdinger, Gottfried Baier, Lukas A. Huber, Gabriele Baier-Bitterlich

**Affiliations:** 1 Division of Neurobiochemistry, CCB-Biocenter, Medical University of Innsbruck, Innsbruck, Austria; 2 Division of Cell Genetics, Department for Pharmacology and Genetics, Medical University of Innsbruck, Innsbruck, Austria; 3 Division of Cell Biology, CCB-Biocenter, Medical University of Innsbruck, Innsbruck, Austria; Indiana University School of Medicine, United States of America

## Abstract

LAMTOR2 (p14), a part of the larger LAMTOR/Ragulator complex, plays a crucial role in EGF-dependent activation of p42/44 mitogen-activated protein kinases (MAPK, ERK1/2). In this study, we investigated the role of LAMTOR2 in nerve growth factor (NGF)-mediated neuronal differentiation. Stimulation of PC12 (rat adrenal pheochromocytoma) cells with NGF is known to activate the MAPK. Pharmacological inhibition of MEK1 as well as siRNA–mediated knockdown of both p42 and p44 MAPK resulted in inhibition of neurite outgrowth. Contrary to expectations, siRNA–mediated knockdown of LAMTOR2 effectively augmented neurite formation and neurite length of PC12 cells. Ectopic expression of a siRNA-resistant LAMTOR2 ortholog reversed this phenotype back to wildtype levels, ruling out nonspecific off-target effects of this LAMTOR2 siRNA approach. Mechanistically, LAMTOR2 siRNA treatment significantly enhanced NGF-dependent MAPK activity, and this effect again was reversed upon expression of the siRNA-resistant LAMTOR2 ortholog. Studies of intracellular trafficking of the NGF receptor TrkA revealed a rapid colocalization with early endosomes, which was modulated by LAMTOR2 siRNA. Inhibition of LAMTOR2 and concomitant destabilization of the remaining members of the LAMTOR complex apparently leads to a faster release of the TrkA/MAPK signaling module and nuclear increase of activated MAPK. These results suggest a modulatory role of the MEK1 adapter protein LAMTOR2 in NGF-mediated MAPK activation required for induction of neurite outgrowth in PC12 cells.

## Introduction

Signaling pathways in eukaryotic cells are often controlled by the formation of specific signaling complexes which are coordinated by scaffold and adaptor proteins. A well-studied signaling pathway is the mitogen-activated protein kinase (MAPK/ERK) cascade, which underlies the regulation of many cellular processes [1]. The discrete dynamics of MAPK activation are believed to be the underlying cause of differences in cellular response [2], [3], [4], [5]. To date, several scaffold proteins have been identified that facilitate MAPK activation in mammalian cells, such as the kinase suppressor of Ras 1 (KSR1) and the MEK1 partner (LAMTOR3/MP1), which is recruited to late endosomes by the adapter protein LAMTOR2/p14 [1], [5], [6], [7]. Besides its role as a scaffold for MAPK signaling, the LAMTOR2/LAMTOR3 complex has been shown to be important for endosomal biogenesis and routing of receptors such as the epidermal growth factor receptor (EGFR) [7], [8].

LAMTOR3 and LAMTOR2 form a heterodimer as part of the larger LAMTOR/Ragulator complex consisting of LAMTOR1 (p18), LAMTOR2 (p14), LAMTOR3 (MP1), LAMTOR4 (C7orf59) and LAMTOR5 (HBXIP), and is required for MAPK and mTOR1 signaling from late endosomes/lysosomes [1], [5], [7], [9], [10], [11], [12], [13], [14]. Depletion of LAMTOR2 was shown to result in mislocalization of LAMTOR3 to the cytoplasm and in defective EGF-mediated MAPK signaling [7], [14]. Deletion of LAMTOR2 also decreases protein stability of the other four LAMTOR components [9], [11].

Early observations in our laboratory indicated that LAMTOR2 [15] is an essential modulator of NGF-mediated differentiation. In view of the critical role of LAMTOR2 for the stability of the entire LAMTOR complex and the controversial role of mTOR1 signaling in neuronal differentiation [16], [17], [18], [19], in this study, we focused on the role of LAMTOR2 in NGF/MAPK-mediated differentiation of PC12 cells. This cell line has been extensively used as a model for investigating NGF-induced signal transduction events because it can mimic NGF-induced survival or differentiation observed in neuronal cells [20].

The aim of the present study was to investigate the role of LAMTOR2/MAPK module in neuronal signaling. We were able to show that LAMTOR2 is a negative regulator for NGF-mediated neurite formation in PC12 cells.

## Materials and Methods

### Reagents

PC12 cells were obtained from LGC Promochem ATCC (Manassas VA, USA). RPMI 1640 medium, L-glutamine, and penicillin/streptomycin were purchased from PAA Laboratories (Vienna, Austria). Horse serum and fetal calf serum were from GIBCO Invitrogen (Vienna, Austria). NGF-β (NGF), EGF, bovine serum albumin (BSA), aprotinin, leupeptin, NaF, NaP-P, Na_3_VO_4_, paraformaldehyde (PFA), and Phalloidin-TRITC were obtained from Sigma (Cologne, Germany). All culture flasks, dishes, and collagen-S type I were from Becton Dickinson (Canaan CT, USA); chamber slides were from NUNC (Rochester NY, USA). Nitrocellulose membrane, Hybond-P PVDF membrane and the enhanced chemiluminescence HRP-substrate (ECL reagent) were obtained from GE Healthcare Biosciences (Uppsala, Sweden). Anti-phospho-p42/44MAPK (ERK1/2), anti-pan-p42/44MAPK and anti-pan-Akt were obtained from Cell Signaling Technology (Danvers, MA, USA). Anti-LAMTOR1/p18 and anti-LAMTOR4/C7orf59 were obtained from Atlas Antibodies (Stockholm, Sweden), anti-LAMTOR3/MP1 and anti-LAMTOR2/p14 were provided by the laboratory of Dr. L. Huber [7], and anti-LAMTOR5/HBXIP was obtained from Santa Cruz (Heidelberg, Germany) Goat anti-rabbit IgG or goat anti-mouse IgG HRP-linked antibodies were from Pierce (Rockford IL, USA). Kits for Bio-Plex phosphoprotein detection/lysis were obtained from Biorad (Vienna, Austria). Anti-EEA1 was purchased from BD Transduction Laboratories (Vienna, Austria) and anti-pan-TrkA from Abcam (Cambridge, UK). Hoechst 33342, goat anti-mouse Alexa 568, goat anti-mouse Alexa 555 and goat anti-rabbit Alexa 488 were obtained from Molecular Probes (Oregon, USA). MOWIOL 4–88 for immunofluorescence studies was from Calbiochem/Merck Millipore (Vienna, Austria). The siGENOME SMART pools for p42/44MAPK and LAMTOR2/p14 and the pool for scrambled non-targeting small interfering RNA (siRNA) duplexes were purchased from Dharmacon (Chicago, IL, USA) and siRNA-resistant human LAMTOR2/p14 ortholog from OriGene (Rockville, MD, USA). Further reagents for transfection were obtained from Amaxa Biosystems (Lonza, Cologne, Germany) and TaqMan Gene Expression Cells-to-CT Kit from Applied Biosystems (Vienna, Austria). All other chemicals were from NeoLab (Migge, Germany) or Applichem (Darmstadt, Germany). Anti-pan GAP-43 clone GAP-7B10, and pharmacological TrkA inhibitor K-252a were obtained from Sigma Aldrich (Vienna, Austria).

### Cell Culture

PC12 cells were grown in RPMI 1640 medium supplemented with 1% Pen/Strep, 1% L-glutamine, 10% horse serum and 5% fetal calf serum at 37°C with 5% CO_2_ on collagen-S type I-coated culture dishes. Cells were subcultured at a density of about 80% or 2 days before starting an experiment. The effects of NGF are mediated by two different cell surface receptors (reviewed in [21]: the receptor tyrosine kinase TrkA [22], [23] and p75NTR (also called p75) [24], [25], both of which are present on PC12 cells [26].

### Post-transcriptional Gene Silencing

Synthetic siRNA directed against p42, p44 MAPK or LAMTOR2/p14 and scrambled non-targeting siRNA duplexes (control siRNA) were used for transient Amaxa Nucleofector-based transfection as described previously [27], [28]. Briefly, approximately 1.5×10^7^ PC12 cells were mixed with nucleofector solution from the “Neuronal Cell Nucleofector kit V” and 1000 nM siRNA. After transfection with the nucleofection device II (program U-029), cells were transferred to prewarmed culture dishes. The transfection efficiency of PC12 cells was 76.25±1.58%. To rule out nonspecific off-target effects of LAMTOR2/p14 siRNA, a human LAMTOR2/p14 ortholog (5 µg) was cotransfected.

### Neurite Outgrowth Assay

Wild type or transfected cells were incubated for various time periods (6–72 h) with NGF or EGF (concentrations according to data in the literature [4] and our own preliminary experiments). Thereafter, pictures were taken of 3–5 independent microscopic fields under blind trial conditions, and neurite-bearing cells were counted. Cells with neurites longer than twice their cell diameter were defined as cells with neurites. Results were calculated and presented either as percentages of neurite-bearing cells [neurite-bearing cells × 100/total cells] or fold of control [% neurite-bearing treated cells/% neurite-bearing control cells]. Neurite length was measured using ImageJ, linking neurite length to the amount of pixels measured.

### Phalloidin Staining

PC12 cells were cultured on chamber slides and treated once with NGF (5 ng/mL) or alternatively pretreated with MEK1-inhibitor PD098059 (50 µM). For studies, cells were generally stained with phalloidin 16 h after incubation. Briefly, cells were washed with 1× phosphate buffered saline (PBS) and fixed for 10 min at room temperature (RT) in 4% PFA. After extensive washing in 1× PBS, cells were permeabilized with 0.5% Triton X-100 for 15 min at RT. Washes followed and cells were stained with 0.2 µM tetramethylrhodamine isothiocyanate-labeled phalloidin (Phalloidin-TRITC) and Hoechst 33342 (10 µg/mL) for 40 min at RT in the dark. To remove unbound phalloidin conjugate, cells were washed several times with 1× PBS. Chamber slides were cover slipped with MOWIOL 4–88. Cells of 3–5 independent microscopic fields were visualized under blind trial conditions under a fluorescence microscope (Zeiss Axioplan2, Austria) equipped with a spot camera (RT-slider 2.3.1 Visitron Systems, Germany) using Hoechst and TRITC filters.

### GAP-43 Staining

Transfected PC12 cells were grown on coated chamber slides or glass coverslips and stimulated with or without NGF (25 ng/ml) for 24 h. After washing the cells in prewarmed 1× PBS, cells were fixed with 4% PFA supplemented with 5% sucrose for 10 min at RT, washed again, permeabilized in ice cold methanol for 2 min and blocked for 30 min with 3% BSA. For labeling, cultures were incubated with monoclonal primary antibody (anti-pan GAP-43 clone GAP-7B10) diluted 1∶1000 in 1% BSA overnight at 4°C. After several washing steps in 1× PBS, incubation for 30 min at RT with the secondary antibody solution (Alexa 555- conjugated goat anti-mouse IgG of F(ab´)_2_ fragments, 1∶1000 in 1% BSA) followed. Cells were covered with MOWIOL 4–88, and pictures were taken under blind trial conditions using a fluorescence microscope (Zeiss Axioplan2, Austria) equipped with a spot camera (RT-slider 2.3.1 Visitron Systems, Germany).

### Colocalization Experiments and Image Analysis

For colocalization analysis, PC12 cells were transfected with control or specific siRNA for LAMTOR2. One day after transfection, cells were put on ice for about 5 min and thereafter left untreated (control) or treated with NGF (200 ng/ml) for various time points (0, 5 and 15 min). After stimulation, cells were washed once in 1× PBS, fixed in 4% PFA for 10 min, permeabilized in 0.2% Triton-X-100 for 2 min and blocked in blocking solution (0.5 g gelatin in 10 ml H_2_O, 2.5 ml 500 mM NH_4_Cl, BSA, 12.5 ml 2× CB (20 mM Pipes [pH 6.8], 300 mM NaCl, 10 mM EGTA, 10 mM glucose, 10 mM MgCl_2_)) for 30 min at RT. Anti-EEA1 (1∶200 in blocking solution) and anti-pan-TrkA (1∶200 in blocking solution) were applied for 2 h at RT in the dark. After extensive washing, secondary antibodies (goat anti-mouse Alexa 568 1:2500 and goat anti-rabbit Alexa 488 1:1000 in blocking solution) were added for 40 min in the dark followed by 3–4 washing steps with 1× PBS. In the last step, Hoechst 33342 (8 µM) was added as nuclear marker. Cells were mounted in MOWIOL 4–88 and documented in one section per cell (at the level of the nucleus) using the Leica TCS SP5 confocal microscope followed by analysis with Huygens Professional 3.7 software (Scientific Volume Imaging, Laapersveld, The Netherlands). Images were deconvolved applying the classical maximum likelihood estimation (CMLE) with the number of iterations set to 100, the quality change threshold to 0.1% and the signal- to-noise ratio varying between 7 and 15 in each channel. Colocalization analysis was performed with the “Colocalization Threshold” plugin of ImageJ. The threshold is determined in two stages and hence the percentage of voxels, which have both channel intensities above threshold, is expressed as percentage of the total number of pixels in the image.

### Cell Lysis and Western Blot Analysis

Prior to experiments, PC12 cells were treated according to previously developed protocols [27], [28], [29], [30]. Untransfected PC12 cells were incubated for 16 h in serum-reduced RPMI medium (RPMI 1640, 1% Pen/Strep, 1% L-glutamine, 0.63% horse serum, 1.25% fetal calf serum) and stimulated. For total cell lysis, cell equivalents (∼6×10^6^ cells) were collected and centrifuged (453 *g*, 5 min, RT), and cell pellets were dissolved in 1× lysis buffer (50 mM Tris [pH 8.5], 1% NP-40, 5 mM EDTA, 50 mM NaCl, 5 mM NaP–P, 5 mM NaF, 5 mM Na_3_Vo_4_, 30 µg/mL aprotinin, and 30 µg/mL leupeptin). The cell suspension was vortexed and kept on ice for at least 20 min. After centrifugation (17300 *g*, 15 min, 4°C), supernatants were transferred to tubes with 4× Laemmli sample buffer (40% glycerin, 240 mM TRIS [pH 6.8], 4% SDS, 0.008% bromophenol blue sodium salt, and 0.2 M mercaptoethanol) and boiled at 95°C for 5 min. For preparing cytoplasmic and nuclear fractions, cells (∼6×10^6^ cells) were harvested after stimulation. The pellet was resuspended in buffer I (10 mM HEPES [pH 7.9], 10 mM KCl, 0.1 mM EDTA, 0.1 mM EGTA, 1 mM DTT, 0.5 mM PMSF), kept on ice for 15 min and 10% NP-40 was added (0.6% final). This was followed by a centrifugation step (2600 *g*, 5 min, 4°C) to separate the cytoplasmic (supernatant) and the nuclear fraction (pellet). After washing the nuclear pellet twice in buffer I, it was resuspended in buffer II (20 mM HEPES [pH 7.9], 0.4 M NaCl, 1 mM EDTA, 1 mM EGTA, 1 mM DTT, 1 mM PMSF) and shaken at 4°C for about 30 min. After centrifugation (15000 *g*, 10 min, 4°C) 4× Laemmli sample buffer (40% glycerin, 240 mM TRIS [pH 6.8], 4% SDS, 0.008% bromophenol blue sodium salt, and 0.2 M mercaptoethanol) was added to the nuclear extracts and boiled at 95°C for 5 min. Extracts were separated on SDS-polyacrylamide gels (SDS-PAGE). Proteins were electroblotted to nitrocellulose or PVDF membranes, which were then blocked in 1× Tris-buffered saline (TBS: 137 mM NaCl, 20 mM Tris-HCl [pH 7.5]) containing 5% skim milk for 1 h at RT and subsequently immunoprobed overnight at 4°C with one of the following antibodies, namely anti-pan-LAMTOR2/p14 or anti-pan-Akt, anti-phospho-p42/44 MAPK or anti-pan-p42/44 MAPK, diluted in 1× Tris-buffered saline containing 0.05% Tween-20 (TBS-T) with 5% BSA. To analyze LAMTOR, 1,3,4,5 total/cytoplasmic cell extracts were prepared and immunoprobed overnight at 4°C with one of the following antibodies, namely anti-pan LAMTOR1/p18, anti-pan LAMTOR3/MP1, anti-pan LAMTOR4/C7orf59 or LAMTOR5/HBXIP. After extensive washing with 1× TBS-T, membranes were further incubated with horseradish peroxidase (HRP)-conjugated goat anti-rabbit IgG or goat anti-mouse IgG (1∶5000 in 5% skim milk in 1× TBS) and identified by a chemiluminescent HRP substrate. For quantification, immunoreactive bands were measured directly by scanning the blots with the Fusion FX7 image acquisition system (Vilber Lourmat).

### Bio-Plex Phosphoprotein Detection Assay

1×10^4^ untransfected and transfected (control, LAMTOR2/p14 siRNA ± human LAMTOR2/p14 ortholog) cells were lysed by adding lysis buffer (Biorad Factor I 1∶250, Factor II 1∶500, PMSF 2 mM) directly to the cells. After 20 min on ice, a centrifugation step (4500 *g*, 20 min, 4°C) followed. The supernatant was diluted in one volume assay buffer and analyzed using the Bio-Plex phosphoprotein detection assay. Briefly, nonmagnetic beads coupled to antibodies against the target protein were added to the filter plate. After washing steps, lysates were added and incubated overnight (gentle shaking at 4°C). This was followed by several washing steps. Biotin-labeled detection antibodies specific for a secondary epitope on the target were added, and after a 30 min incubation step at RT, streptavidin-PE was used for detection with the Bio-Plex system.

### Quantitative Real-time RT-PCR

Cells-to-CT technology enables reverse transcription of lysates from cells without preceding RNA purification. Briefly, transfected (LAMTOR2/p14 siRNA and control siRNA) cells were washed in cold 1× PBS. Thereafter, lysis solution (1∶100) was added and after 5 min incubation at RT, cell lysis was stopped by adding stop solution. Reverse transcription and real-time PCR analysis was carried out directly thereafter. Expression analysis was done in duplicates on an ABI PRIM 7000 Sequence Detection System with TaqMan gene-expression assays. To determine the relative quantification of a target gene (LAMTOR2) in comparison to a reference gene (GAPDH), the mathematical model presented by PE Applied Biosystems (Perkin Elmer) and described by Pfaffl [31] was used: 2^−^∧∧^ct^ method.

### Statistical Analysis

Results are presented as means ± SEM of the indicated number of independent experiments. The SPSS 19.0 statistics program was applied for analysis of experiments. The unpaired two-tailed t-test was used to compare two independent groups. *P*-values <0.05 were considered statistically significant (*P<0.05, **P<0.01, ***P<0.001). When more than two groups were compared, data were analyzed using one-way ANOVA followed by Dunnett’s multiple comparison test. *P*-values <0.05 were considered statistically significant (*P<0.05, **P<0.01, ***P<0.001).

## Results and Discussion

**Figure 1:**
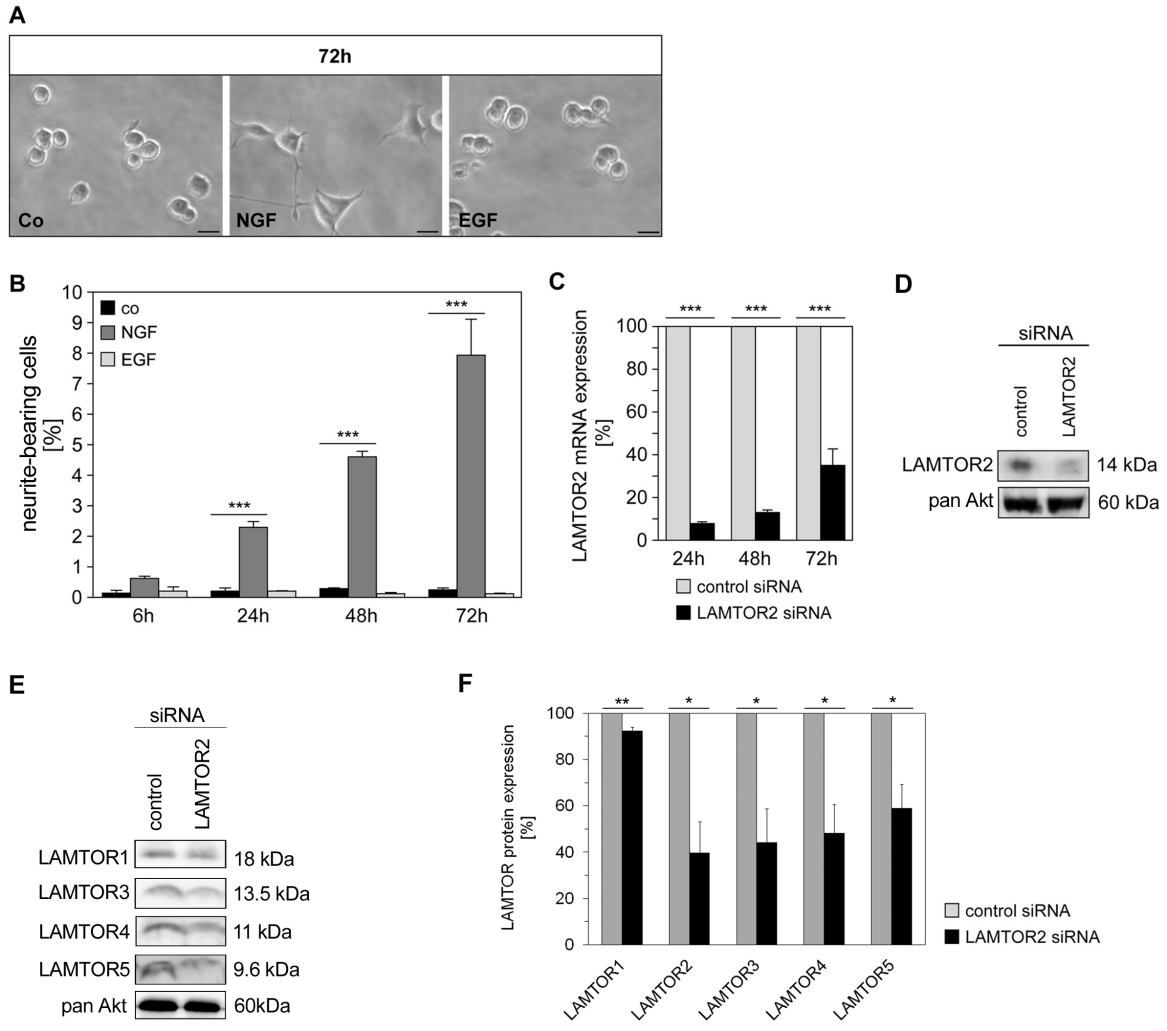
LAMTOR2 adapter protein modulates NGF-mediated differentiation. (A, B) PC12 cells were stimulated with either NGF (25 ng/ml) or EGF (100 ng/ml). Neurite outgrowth was examined in 3–5 independent microscopic fields (72 h after stimulation shown in (A)). Percentages of neurite outgrowth (numbers reflecting neurite-bearing cells) after various time points (6 h–72 h) were calculated, counting 500–700 cells for each time point, and are depicted in (B). Microscopic images are representative of 3 independent experiments. Values represent the mean ± SEM, n = 3 (number of experiments). Scale bar = 20 µm. Differences were analyzed using one-way ANOVA followed by Dunnett’s multiple comparison test: ***p<0.001. (C) PC12 cells were transfected with control or specific LAMTOR2 siRNA, and mRNA expression levels were examined after 24 h, 48 h and 72 h by quantitative real-time RT-PCR; values represent the mean ± SEM, n = 3–5. Differences were analyzed using unpaired two-tailed t-test: ***p<0.001. (D) LAMTOR2 protein expression levels were shown by western blotting technique 48 h after transfection. Blot is representative of 3 independent experiments. (E) PC12 cells transfected with control or specific siRNA for LAMTOR2 were analyzed for protein expression of the other members of the LAMTOR complex, LAMTOR1 (p18), LAMTOR3 (MP1), LAMTOR4 (C7orf59) and LAMTOR5 (HBXIP) at 48 hours. Blots are representative of 3 experiments. (F) LAMTOR downregulation (%) was quantified and differences analyzed using unpaired two-tailed t-test: LAMTOR1 (p18; 7.63±1.50% reduction, **p<0.01), LAMTOR2 (p14; 60.25±13.54% reduction, *p<0.05), LAMTOR3 (MP1; 55.91±14.68% reduction, *p<0.05), LAMTOR4 (C7orf59; 51.85±12.52% reduction, *p<0.05) and LAMTOR5 (HBXIP; 41.06±10.33% reduction, *p<0.05).

When PC12 cells were incubated with the two growth factors EGF or NGF, only the latter efficiently supported the development of neurites, which was monitored over a time period of three days in PC12 cells (7.94±1.22% percentage of neurite-bearing cells) (Fig. 1A and B). This result stands in line with previous observations in PC12 cells representing a model of choice to compare the signaling of growth factors such as NGF and purine nucleosides related to differentiation processes and of mitogenic growth factors such as EGF [27], [32], [33].

Based on our earlier observation indicating that the adapter protein LAMTOR2 [15] is an essential player in NGF-mediated differentiation, we further focused on the role of the MAPK/LAMTOR module. Transfection of PC12 cells with synthetic specific siRNA for LAMTOR2 resulted in efficient knockdown (e.g. 87.78±7.86% LAMTOR2 mRNA and 60.25±13.54% LAMTOR protein expression at 48 hours), as shown by analysis of LAMTOR2 mRNA (Fig. 1C) and protein expression levels (Fig. 1D and F) in PC12 cells. This also resulted in reduced protein expression of the other members of the LAMTOR complex, LAMTOR1 (p18; 7.63±1.50% reduction), LAMTOR3 (MP1; 55.91±14.68% reduction), LAMTOR4 (C7orf59; 51.85±12.52% reduction) and LAMTOR5 (HBXIP; 41.06±10.33% reduction) at 48 hours (Fig. 1E and F). Knockdown of LAMTOR2 also decreased protein stability of the other four LAMTOR components. This result is in agreement with previous reports on destabilization of the entire LAMTOR/Ragulator complex following inhibition of LAMTOR2 [9], [11].

**Figure 2:**
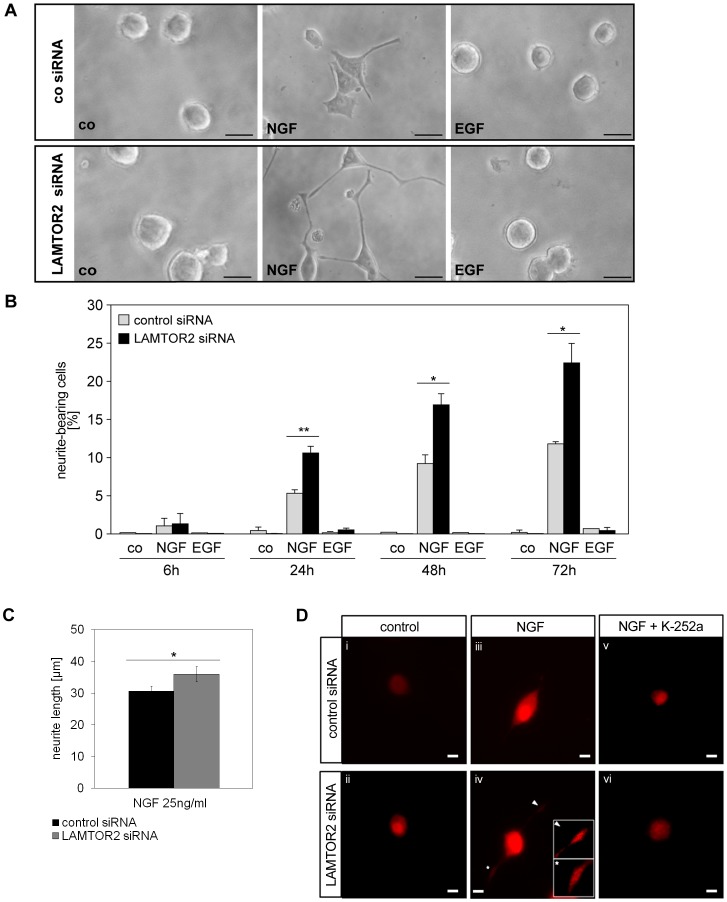
LAMTOR2 knockdown increases neurite formation of NGF- but not EGF-treated PC12 cells. (A, B) PC12 cells, transfected either with control or specific LAMTOR2 siRNA, were left untreated or stimulated with NGF (25 ng/ml) or EGF (100 ng/ml). Neurite outgrowth was examined in 3–5 independent microscopic fields (72 h after stimulation shown in (A)). Percentages of neurite-bearing cells after various time points (6 h –72 h) were calculated and are depicted in (B). Microscopy images are representative of 3 independent experiments. Values represent the mean ± SEM, n = 3. Scale bar = 20 µm. Differences were analyzed using unpaired two-tailed t-test: *p<0.05. (C) Analyses of neurite length in µm. Values represent the mean ± SEM, n = 9. Differences were analyzed using unpaired two-tailed t-test: *p<0.05. (R1-1.2a). (D) The expression of the plasticity protein GAP-43 was analyzed in control- (i, iii and v) and LAMTOR2 knockdown (ii, iv and vi) PC12 cells that were stimulated with (iii–vi) and without NGF (unstimulated control i-ii) in the presence (v-vi) and absence (i-iv) of the TrkA inhibitor K-252a. Icons in iv (higher magnification of GAP-43 stained neurites). Microscopy images are representative of 4 independent experiments. Scale bar = 10 µm.

In a next step, we investigated the effect of LAMTOR2 knockdown on neurite formation of PC12 cells. To our surprise, knockdown of LAMTOR2 did not inhibit neurite formation but significantly augmented both NGF-mediated neurite outgrowth (Fig. 2A and B), and neurite length (Fig. 2C). In parallel, the expression of the plasticity protein GAP-43 was analyzed in control and LAMTOR2 knockdown PC12 cells that were stimulated with and without NGF in the presence and absence of the TrkA inhibitor K-252a (Fig. 2D). GAP-43 expression was enhanced in cells transfected with LAMTOR2 siRNA as compared to cells transfected with control siRNA, both in unstimulated (Fig. 2Di and ii) and in NGF-stimulated PC12 cells (Fig. 2D iii and iv). Addition of K-252a resulted in a drastic downregulation of GAP-43 expression levels in control- and LAMTOR2–siRNA transfected NGF-stimulated PC12 cells (Fig. 2D v and vi).

**Figure 3:**
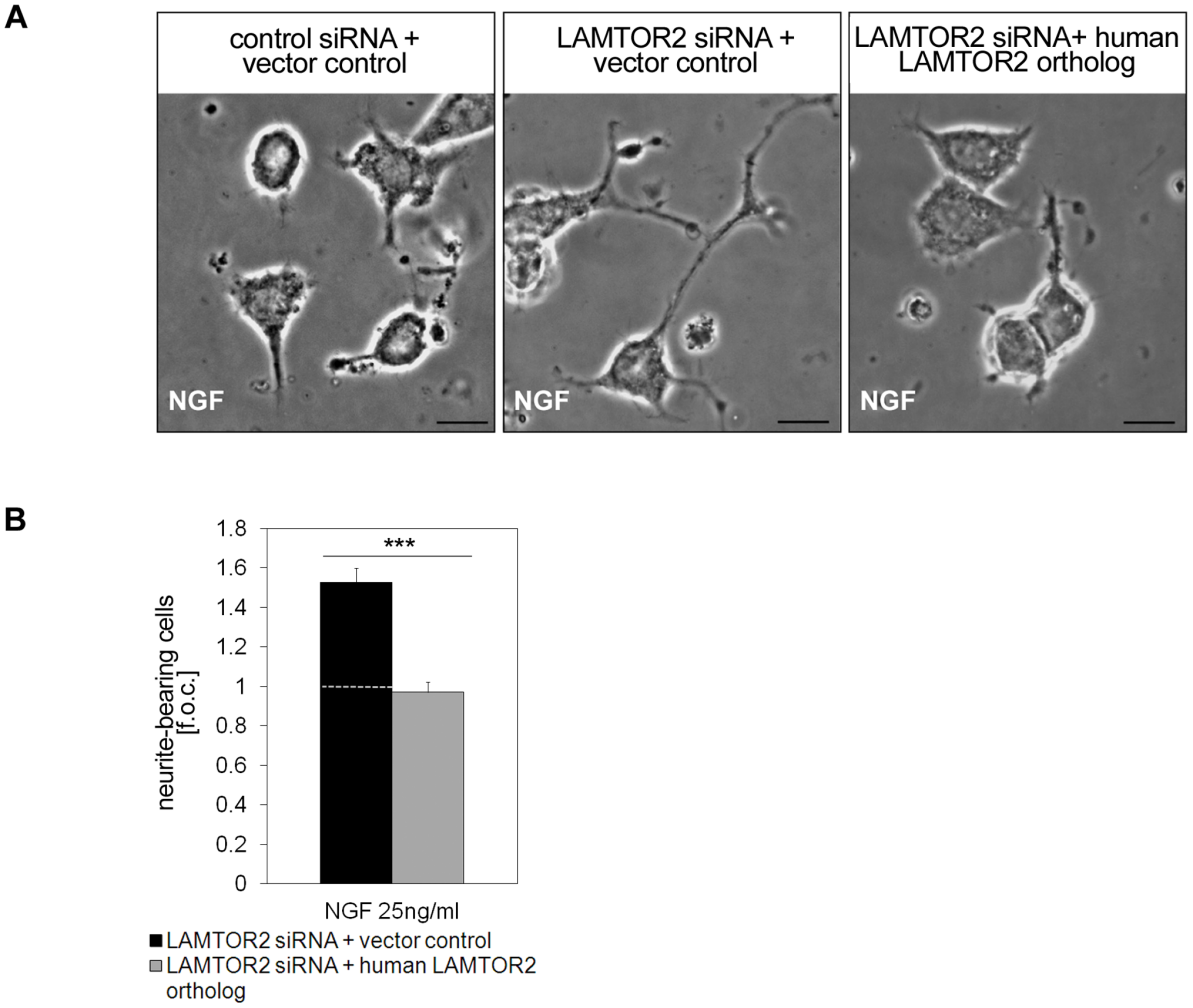
LAMTOR2 phenotype is rescued with a human LAMTOR2 ortholog. (A, B) Control or LAMTOR2 siRNA silenced cells were cotransfected with a human LAMTOR2 ortholog and stimulated with NGF (25ng/ml). Pictures of 3–5 independent microscopic fields were taken after 16 h (A) and neurite-bearing cells were calculated as fold of control (f.o.c. control value was 22.45±1.39% neurite-bearing cells) in (B). Pictures are representative of 4 independent experiments. Values represent the mean ± SEM, n = 4. Scale bar = 20 µm. Differences were analyzed using unpaired two-tailed t-test: ***p<0.001.

Ectopic expression of a siRNA-resistant LAMTOR2 ortholog (expression was checked by western blotting, B. T, data not shown) reversed this phenotype (Fig. 3A and B), also for neurite length (data not shown), ruling out nonspecific off-target effects of the LAMTOR2 siRNA approach.

**Figure 4:**
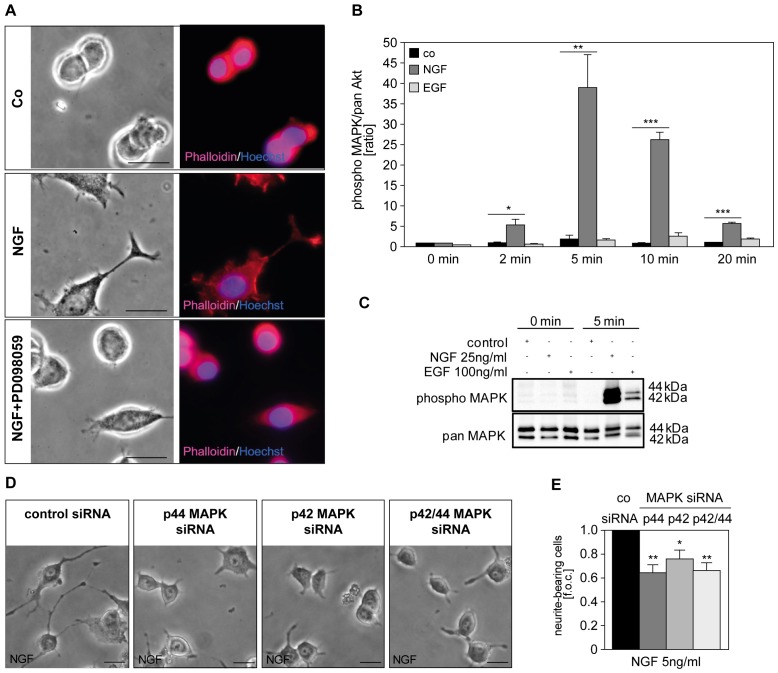
NGF-induced neurite formation depends on the activation of p42/44 MAPK. (A) Cells left untreated (co), stimulated with NGF (25 ng/ml) or alternatively with NGF (25 ng/ml) and PD098059 (50 µM) for 16 h and were stained with Phalloidin-TRITC and Hoechst 33342. Pictures were taken of 3–4 independent microscopic fields and are representative of 3 experiments. Scale bar = 20 µm. (B, C) PC12 cells were stimulated with either NGF (25 ng/ml) or EGF (100 ng/ml) and activation of p42/44 MAPK was measured by Bio-Plex phosphoprotein detection assay at various time points (0 min –20 min). Differences were analyzed using one-way ANOVA followed by Dunnett’s multiple comparison test: *p<0.05, **p<0.01, ***p<0.001 (B). Total cell lysates were analyzed with western blotting done in parallel for 0 and 5 min. (D, E) PC12 cells were transfected with control or specific siRNA for MAPK. After 72 h in culture, cells were stimulated with NGF (5 ng/ml). Additional 3 d pictures were taken of 3–5 microscopic fields (D) and neurite-bearing cells are expressed as fold of control (f.o.c.; control value was 2.16±0.53% neurite-bearing cells) in (E). Pictures are representative of 5 independent experiments. Values represent the mean ± SEM, n = 5. Scale bar = 20 µm. Differences were analyzed using one-way ANOVA followed by Dunnett’s multiple comparison test: *p<0.05, **p<0.01.

Due to the tight connection of LAMTOR2 and p42/44 MAPK, we wanted to analyze the role of MAPK activation in NGF-mediated differentiation. First we approached this question by using the pharmacological inhibitor of MEK1 (PD098059) in our cell system. We observed that PD098059 significantly blocked NGF-mediated differentiation (Fig. 4A). Activity of p42/44 MAPK was significantly augmented following stimulation with NGF, as shown by Bio-Plex phosphoprotein detection (Fig. 4B) and western blot analysis (Fig. 4C). In agreement with this, knockdown of MAPK to the level of about 30% protein expression [27] induced inhibition of NGF-mediated neurite formation (Fig. 4D and E). The effects of siRNA against p44 and p42, however, were not additive. There are several possible explanations for this phenomenon. The results may reflect a non-redundant and isoform-selective role of both p42 and p44 MAPK in NGF-induced neurite formation. Of note, the question whether p42 and p44 MAPK are functionally redundant is still a matter of debate. Human p42 and p44 MAPK are 84% identical in sequence, are regulated similarly, contribute to intracellular signaling by phosphorylating a largely common subset of substrates and share many, if not all, functions [34], [35]. However, Yao et al. reported that p42 and p44 MAPK are not functionally redundant [36]. Alternatively, the observed result may be explained by the haplosufficiency of either p42 and p44 MAPK following siRNA-mediated knockdown, as the remaining leftover of about 30% protein kinase allow, in part, an almost normal cellular function.

**Figure 5:**
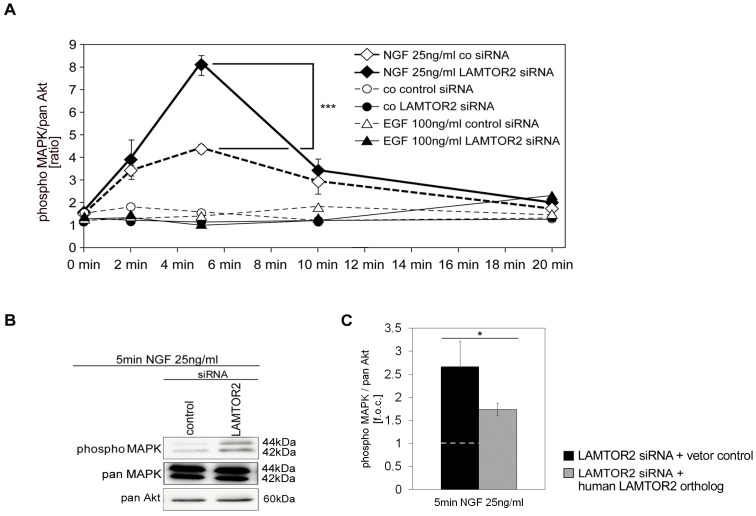
SiRNA-mediated knockdown of LAMTOR2 scaffold protein leads to increased NGF- mediated MAPK activation. (A, B) PC12 cells were transfected with control or specific siRNA for LAMTOR2. After 24 h, cells were left untreated (co) or stimulated with either NGF (25 ng/ml) or EGF (100 ng/ml). After various time points (0, 2, 5, 10, 20 min), activation of p42/44 MAPK was measured with the Bio-Plex phosphoprotein detection assay. Values represent the mean ± SEM, n = 3. Differences were analyzed using unpaired two-tailed t-test: ***p<0.001. (B) In parallel, after 5 min, total cell lysates were analyzed by western blotting technique. (C) Cells were transfected with LAMTOR2 siRNA plus control vector or plus a human LAMTOR2 ortholog. After stimulation with NGF (25 ng/ml) for 5 min Bio-Plex phosphoprotein detection assay was carried out and p42/44 MAPK activation expressed as fold of control (f.o.c. control value was 4.57±0.67%). Values represent the mean ± SEM, n = 7. Differences were analyzed using unpaired two-tailed t-test: *p<0.05.

We were therefore interested to find out whether LAMTOR2 may directly regulate NGF-mediated MAPK activity. PC12 cells were transfected with siRNA to specifically knockdown LAMTOR2 protein expression. Lysates of PC12 cells were then tested for the activation of MAPK. Mechanistically, LAMTOR2 siRNA treatment significantly enhanced NGF-dependent MAPK activities (Fig. 5A). This result was confirmed by western blot analysis of phospho MAPK as compared to pan MAPK/pan Akt (Fig. 5B). These effects were again reversed upon expression of the siRNA-resistant LAMTOR2 ortholog (Fig. 5C).

**Figure 6:**
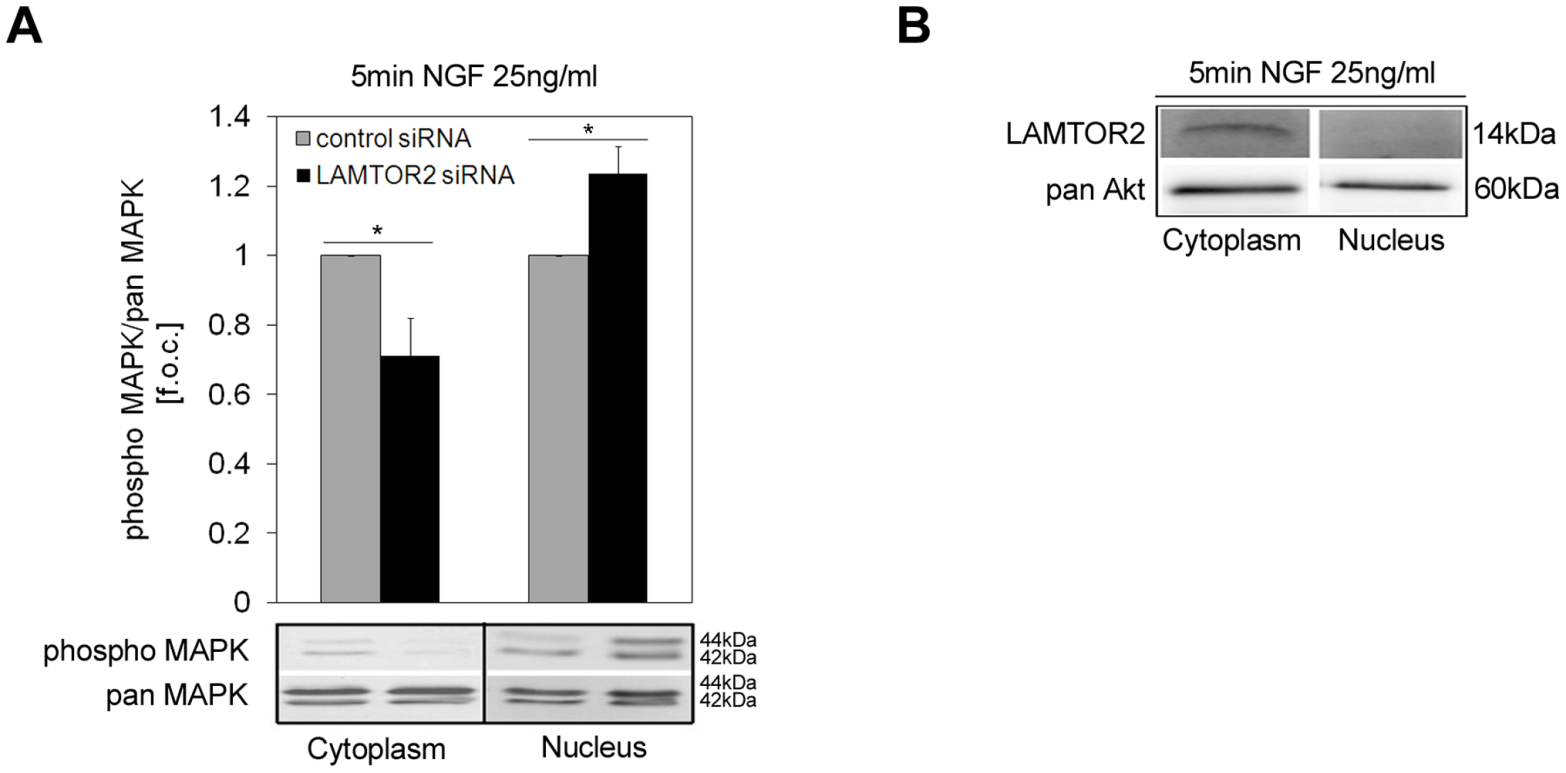
Active MAPK and LAMTOR2 distributions in cellular fractions. PC12 cells were transfected with control or specific siRNA for LAMTOR2. After 24(25 ng/ml) for 5 min and lysed for cytoplasmic and nuclear fractions. Fractions were separated and immunoblotted. (A) MAPK activation was quantified after scanning the immunoreactive bands with the Fusion FX7 image acquisition system (Vilber Lourmat). Values represent the means ± SEM, n = 5. Differences were analyzed using unpaired two-tailed t-test: *p<0.05. (B) LAMTOR2 and pan Akt protein expression was analyzed in cytoplasmic and nuclear fractions. Blots are representative of 4 independent experiments.

In addition, the distribution of active MAPK as compared to pan MAPK, LAMTOR2 and pan Akt in the cytoplasmic and nuclear fraction were analyzed after NGF stimulation. Interestingly, we observed that LAMTOR2 knockdown decreased MAPK activity in the cytoplasm by 28.87±10.85%, whereas active MAPK in the nucleus was increased by 23.57±7.94% (Fig. 6A). In contrast to the even distribution of Akt, LAMTOR2 protein expression was predominant in the cytoplasmic fraction (Fig. 6B).

**Figure 7:**
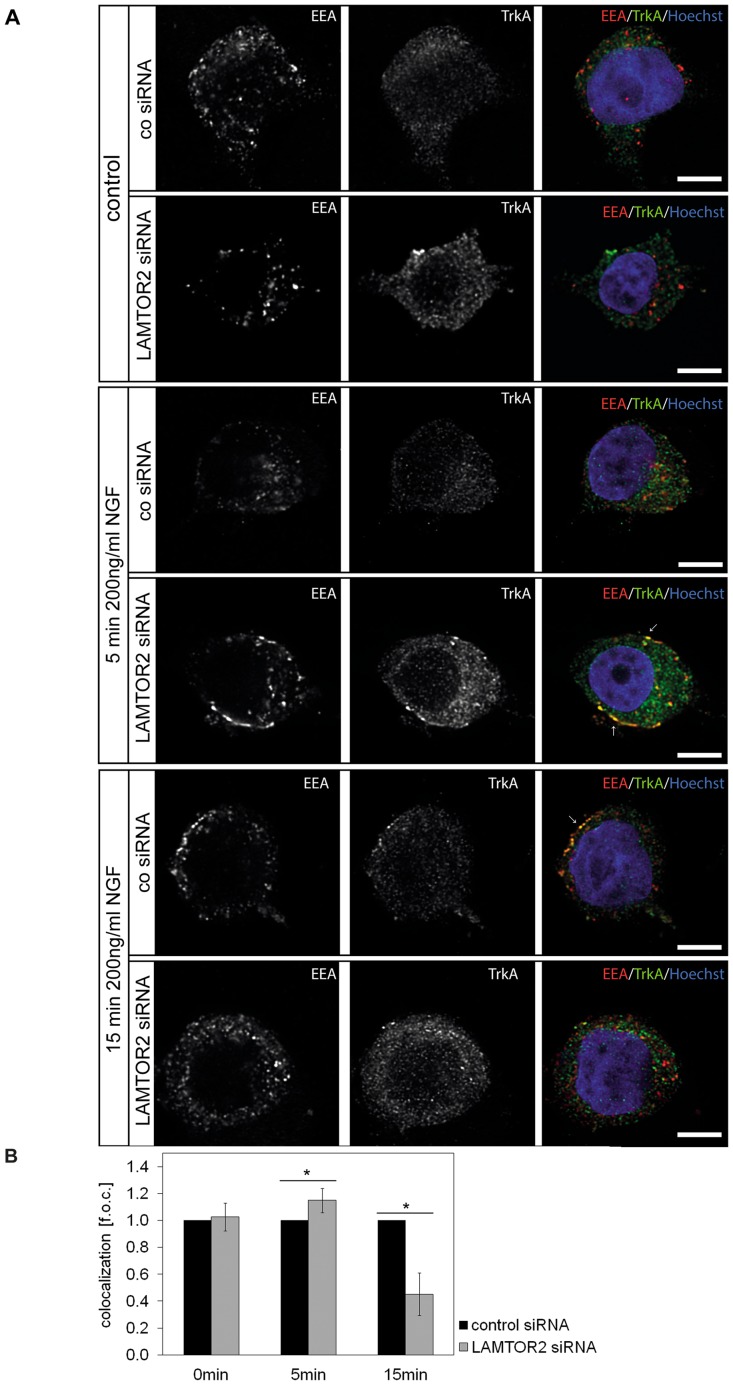
LAMTOR2 is involved in endosomal trafficking. (A, B) PC12 cells were transfected with control or specific siRNA for LAMTOR2. After 24 h, cells were stimulated with NGF (200 ng/ml) for various time points (0 = control, or 5 and 15 min), fixed and stained for EEA1 (early endosomes), pan-TrkA and Hoechst (nucleus). EEA 1 is shown in red and pan-TrkA in green. Colocalization of EEA1 and pan-TrkA is shown in yellow (arrows). Scale bar = 10 µm. Colocalization tests were performed as described in material and methods section. Colocalization in LAMTOR2 knockdown cells is expressed as fold of control (f.o.c.; control values in control siRNA transfected cells: 0 min 20.99±8.55%, 5 min 22.74±7.94%, 15 min 24.94±9.04%). Values represents the means ± SEM, n = 3–5. Differences were analyzed using unpaired two-tailed t-test *p<0.05.

Next, we raised the question whether LAMTOR2 was involved in endosomal trafficking of the NGF receptor TrkA. PC12 cells were transfected with control or siRNA specific for LAMTOR2. Cells were then stimulated with NGF, and colocalization of endosomes and TrkA receptor analyzed. Studies of intracellular trafficking of the NGF receptor TrkA revealed a fast (5 min) colocalization with early endosomes, which was enhanced by 19.39±6.97% by LAMTOR2 siRNA. Yet, after 15 minutes, a significant decrease (44.45±15.79%) of TrkA receptor colocalization with early endosomes was observed (Fig. 7A and B). Concomitant with this result, LAMTOR2 knockdown decreased colocalization of TrkA with late endosomes (B.T. unpublished observation). Due to the abnormal, peripheral distribution of late endosomes in LAMTOR2 knockdown cells, we did not pursue this line of research. Inhibition of LAMTOR2 and concomitant destabilization of the remaining LAMTOR complex apparently leads to a faster release of the TrkA/MAPK signaling module and nuclear increase of activated MAPK.

Our results are in agreement with previous findings [37] that, in fibroblasts obtained from patients suffering from immunodeficiency syndrome caused by deficiency of LAMTOR2, the late endosomes were no longer concentrated in the perinuclear area, but redistributed to the cell periphery, suggesting a role of LAMTOR2 in the control of late endosomal compartment configuration. In LAMTOR2 knockout mouse embryonic fibroblasts (MEFs), late endosomes, multivesicular bodies (MVBs) and lysosomes, but not early endosomes were displaced to the cell periphery [7], [8]. Interestingly, MVBs and lysosomes were also displaced to the cell periphery in MEK−/− MEFs suggesting that the proper positioning of late endosomes require LAMTOR2-LAMTOR3-MEK1 signaling [8]. These data also indicate an exciting link between MAPK signal transduction, organelle biogenesis and intranuclear transport. In a future study, it will be interesting to identify the interplay of NGF receptors and the LAMTOR2-MAPK-MEK1 module. Data presented in this study are in line with previous findings [38], providing evidence that TrkA predominantly recycles back to the cell surface after ligand treatment, whereas TrkB is predominantly sorted to the degradative pathway. A further goal will be the development of a primary nerve cell model. While PC12 cells may be maintained without nerve growth factor (NGF) in culture and the role of NGF in PC12 cell differentiation is clear and reproducible [3], [20], [33], ‘developing sympathetic neurons’ largely depend on NGF for survival and die by apoptosis after NGF withdrawal [39]. For this reason, the TrkA receptor was also called a “dependence receptor,” (reviewed in [40]). Biochemical analysis of the actions of NGF upon primary peripheral neurons has often been hampered by the lack of a variety of neuronal cell models responsive to NGF, but do not require it for survival and also because it is difficult to obtain large numbers of sympathetic neurons for *in vitro* studies [39]. Furthermore, in a primary neuron model the development of sympathetic neurons might be critically regulated by two neurotrophins NT3 and NGF, acting through a common receptor TrkA, as reported earlier [41]. For these reasons, whether the results of the present study using PC12 cells can be extrapolated to primary neurons remains to be investigated.

## Conclusion

Taken together, our data clearly identify a modulatory role of the MEK1 adapter protein LAMTOR2 in NGF-mediated MAPK activation kinetics and neurite outgrowth induction of PC12 cells. Of note, stimulation with EGF significantly enhanced neither neurite formation nor MAPK activity in our system. The MEK1/MAPK pathway constitutes an essential element in NGF-mediated differentiation, shown by pharmacological inhibition of MEK1 with PD098059 and siRNA-mediated knockdown of MAPK. Knockdown of LAMTOR2 unexpectedly led to a positive feedback coupling through the upregulation of MAPK activation and to an intensified differentiation process. Based on our own data and data from other studies [9], [11], we speculate that knockdown of LAMTOR2 unleashes the NGF receptor signaling pathway from late-endosomal degradation, likely via downregulation of the LAMTOR/Ragulator complex thereby allowing a prolongation of the MAPK signal and increased nuclear entry of active MAPK, which favors the differentiation signal.
